# Endovascular aortic repair endograft limb misdeployment salvaged with laser fenestration

**DOI:** 10.1016/j.jvscit.2025.101874

**Published:** 2025-06-09

**Authors:** Mira T. Tanenbaum, Andrea E. Klein, Jacqueline L. Babb, Mirza S. Baig, Melissa L. Kirkwood, Carlos H. Timaran

**Affiliations:** a Department of Surgery, George Washington University Hospital, Washington, DC; b Division of Vascular and Endovascular Surgery, Department of Surgery, University of Texas Southwestern Medical Center, Dallas, TX; c Orlando Health Heart and Vascular Institute, Orlando, FL

**Keywords:** Abdominal aorta, EVAR, Iliac artery, Laser fenestration

## Abstract

Endovascular aortic aneurysm repair (EVAR) offers a minimally invasive treatment approach for abdominal aortic aneurysms (AAAs). Iliac limb misdeployment outside of the gate is a rare complication that traditionally requires open conversion. We describe two cases of limb misdeployment salvaged with *in situ* laser fenestration (ISLF). An ISLF was created through the misplaced iliac limb into the aneurysm sac, allowing for subsequent gate cannulation. A new limb was then deployed from the gate through the fenestration into the iliac artery. These cases demonstrate that ISLF through a misdeployed EVAR limb is a novel bail-out endovascular technique that circumvents open surgery and restores the aortoiliac anatomic continuity of the repair.

The management of abdominal aortic aneurysms (AAAs) has undergone significant changes with the advent of advanced techniques for endovascular aortic aneurysm repair (EVAR).^1^^,^^2^ These advancements significantly reduce the perioperative and postoperative morbidity and mortality associated with traditional open aneurysm repair, making EVAR the preferred approach for most patients.^3^ One technical challenge during EVAR is the cannulation of the contralateral gate.^4^ Misdeployment of the iliac limb outside of the gate can lead to devastating consequences. Historically, the management of such misdeployments required conversion to open repair or femoro-femoral bypass creation.^5^ However, the evolution of endovascular techniques has led to the development and adoption of innovative approaches to address anatomic limitations or intraprocedural complications. One such advancement is *in situ* laser fenestration (ISLF), which involves using laser energy to create fenestrations or perforations in vascular structures or endografts. ISLF has gained prominence in left subclavian artery revascularization, renovisceral vessel incorporation, and internal iliac artery preservation during complex thoracic and abdominal endovascular repair, with promising short-term outcomes and low rates of early mortality or major adverse events.^6^^,^^7^

Despite the promise of ISLF, its application in the management of limb misdeployment during EVAR has not been previously reported. Research on endovascular techniques for addressing this complication remains limited, given its low frequency. However, the potential benefits of ISLF in salvaging limb misplacement highlight the importance of exploring its utility in endovascular surgery. This report describes two cases of EVAR limb misdeployment that were successfully salvaged using ISLF with the goal of providing insights into the feasibility and efficacy of this innovative approach in managing complications encountered during EVAR. The patients described provided consent for the report of their cases and associated images.

## Case reports

**Fig 1:**
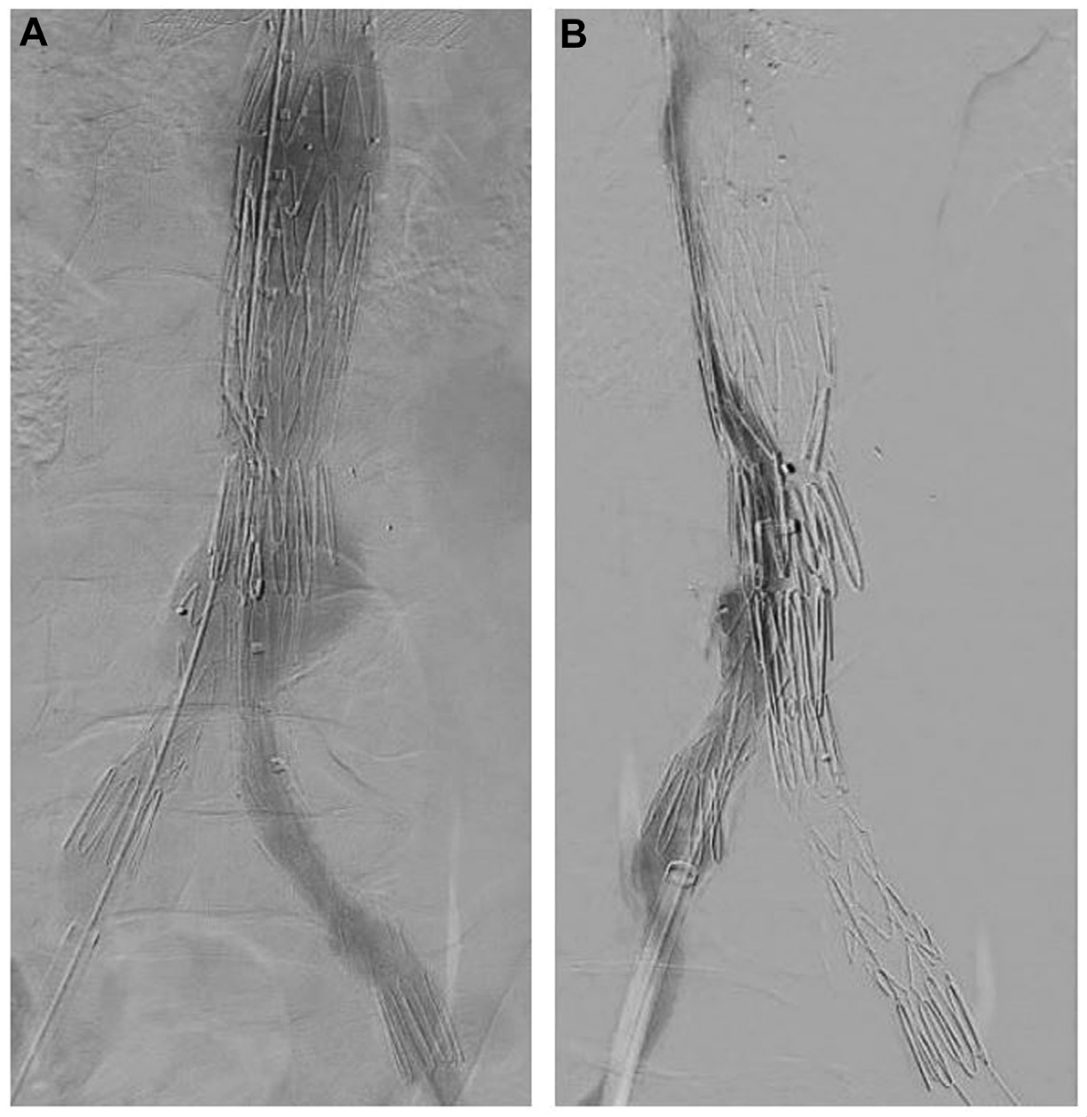
Initial completion fluoroscopy demonstrated no antegrade flow via the right iliac limb **(A)** with selective angiography of the misdeployed limb demonstrating occlusion of the proximal limb with no filling of the endograft main body **(B)**.

**Fig 2:**
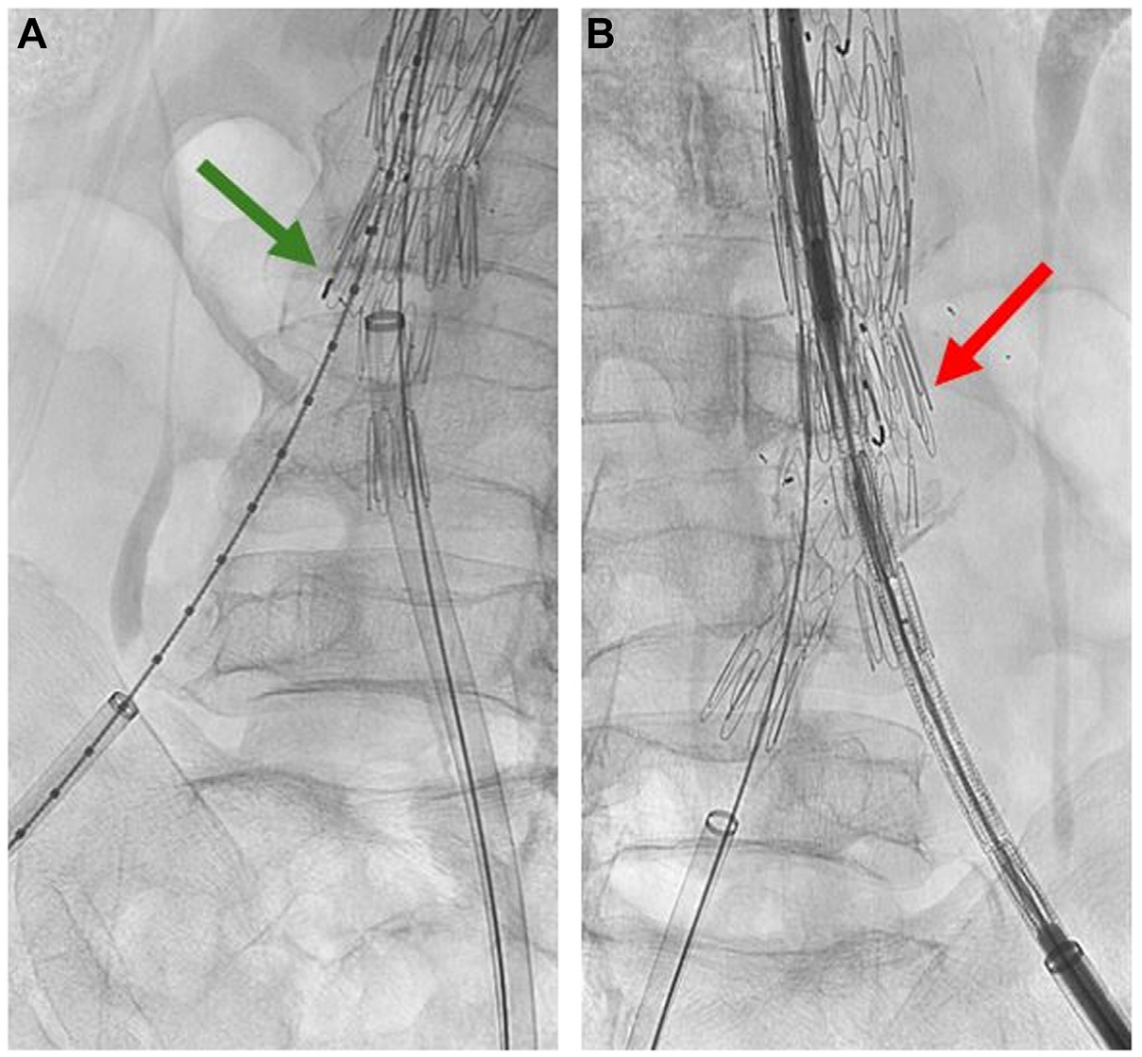
Fluoroscopic images from two different oblique angles of the contralateral gate. On a left anterior oblique view **(A)**, the gate, identified by the *green arrow*, appears to be cannulated. However, in a right anterior oblique view **(B)**, the gate, identified by the *red arrow*, appears unoccupied.

**Fig 3:**
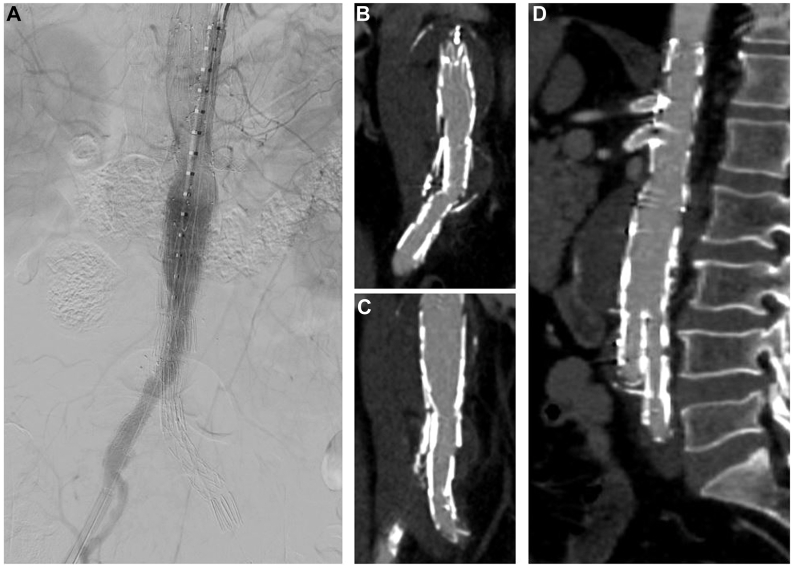
Completion angiography demonstrates antegrade right lower extremity flow via the right iliac limb deployed through the *in situ* laser fenestration (ISLF) **(A)**. On postoperative computed tomography angiography (CTA), both the right iliac limb **(B)** and the left iliac limb **(C)** had inline flow with successful exclusion of the aneurysm sac **(D)**.

The first patient was a 63-year-old male with a 5.6-cm juxtarenal AAA who was recommended to undergo patient-specific company-manufactured fenestrated EVAR as part of a physician-sponsored investigational device exemption (IDE #G140108, NCT #02266719) (Cook Medical, Inc). A narrow distal calcified aorta was evident, but it did not appear to limit the use of a standard bifurcated device. The procedure was performed using bilateral percutaneous access. The distal bifurcated device was deployed after the fenestrated component, and the contralateral gate was cannulated in the standard fashion. Gate cannulation was confirmed on intravascular ultrasound (IVUS), although aortic angulation and a narrow distal aortic lumen limited IVUS visibility and certainty of the cannulation. Other methods to confirm contralateral gate catheterization, such as the ‘ballerina’ maneuver with an Omni-flush or pigtail catheter and/or balloon inflation, were not attempted, given the compression of the contralateral gate and the limited visibility as mentioned above. The contralateral right iliac limb was deployed under fluoroscopic guidance. Completion angiogram revealed that the right limb was misplaced outside of the gate with no antegrade flow to the right leg (Fig 1, *A*). Selective angiography of the misdeployed right limb showed occlusion of the proximal limb without filling of the aneurysm sac (Fig 1, *B*). On close inspection of the lateral fluoroscopy images, it was apparent that the gate had not been successfully cannulated. Rather, the wire and contralateral limb were passed between the infrarenal bifurcated device and the fenestrated endograft with significant compression of the proximal limb between these devices (Fig 2). To avoid the need for an adjunct surgical procedure, we elected to create an ISLF to rectify this mistake. A cone-beam computed tomography (CT) scan was performed to assess the location of the misplaced limb in relation to the aortic aneurysm sac. A steerable sheath was advanced from the right femoral access into the misdeployed limb and directed toward the large aortic aneurysm sac with fusion guidance and the previously obtained cone-beam CT as reference. Using a 2-mm Excimer laser (Spectranetics Corp, Philips Holding USA Inc), a fenestration was created through the iliac limb into the aneurysm sac. A 0.014-in wire was advanced through the fenestration into the aneurysm lumen, and the fenestration was serially dilated using angioplasty balloons. The contralateral gate was ultimately cannulated using a 4 Fr Bern catheter and standard guidewire. Balloon expandable stents (VBX, W.L Gore & Associates) were deployed from the gate through the fenestration into the native right common iliac artery, which re-established inline flow to the right leg (Fig 3, *A*). Postoperative imaging revealed exclusion of the aneurysm sac and patent iliac limbs with antegrade perfusion to both lower extremities (Fig 3, *B*).

**Fig 4:**
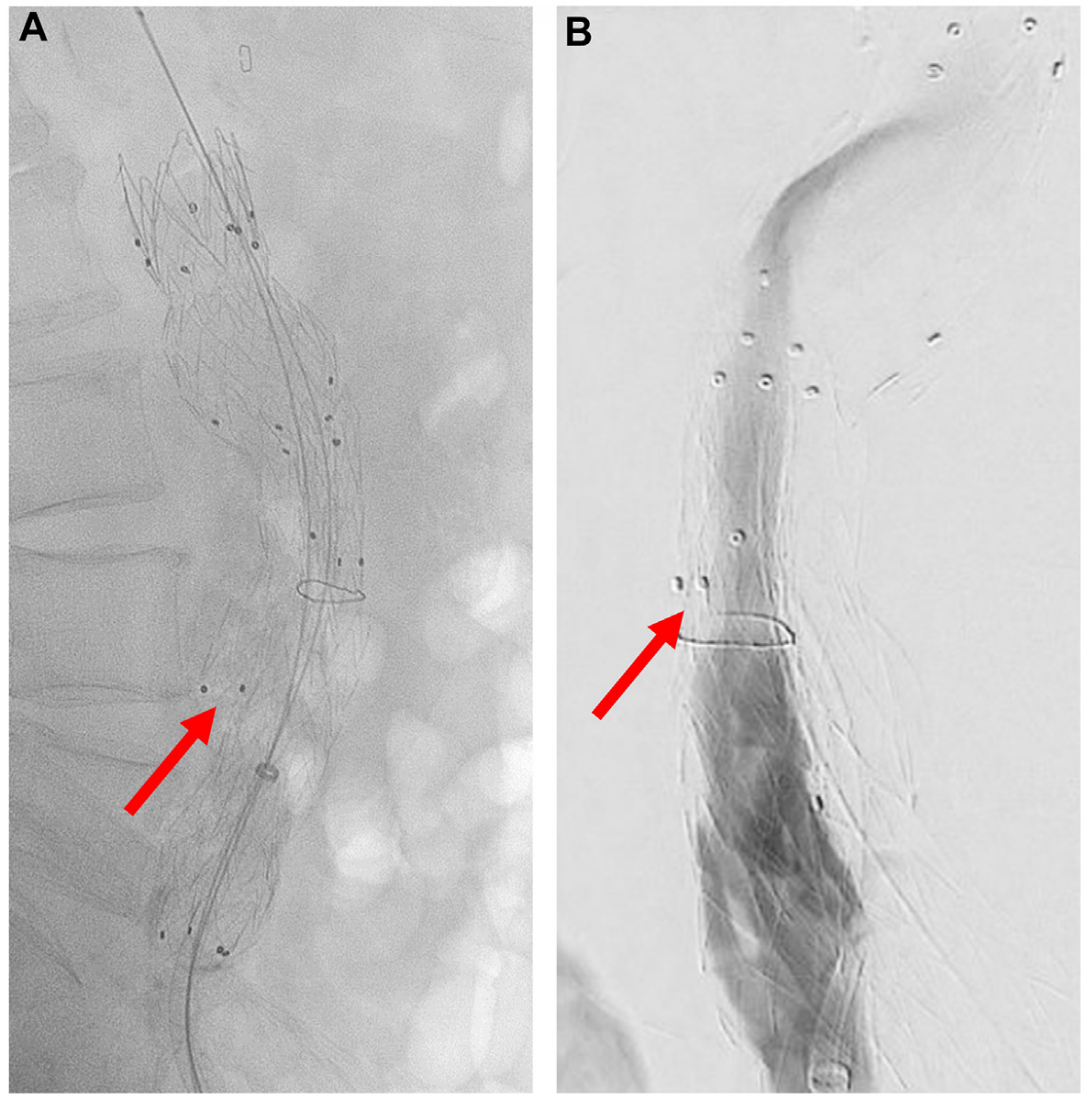
Initial angiography of the right iliac limb demonstrated that it had been misdeployed **(A)**, leading to a type III endoleak from the unoccupied ipsilateral gate (indicated by *red arrows*) off the main body endograft **(B)**.

**Fig 5:**
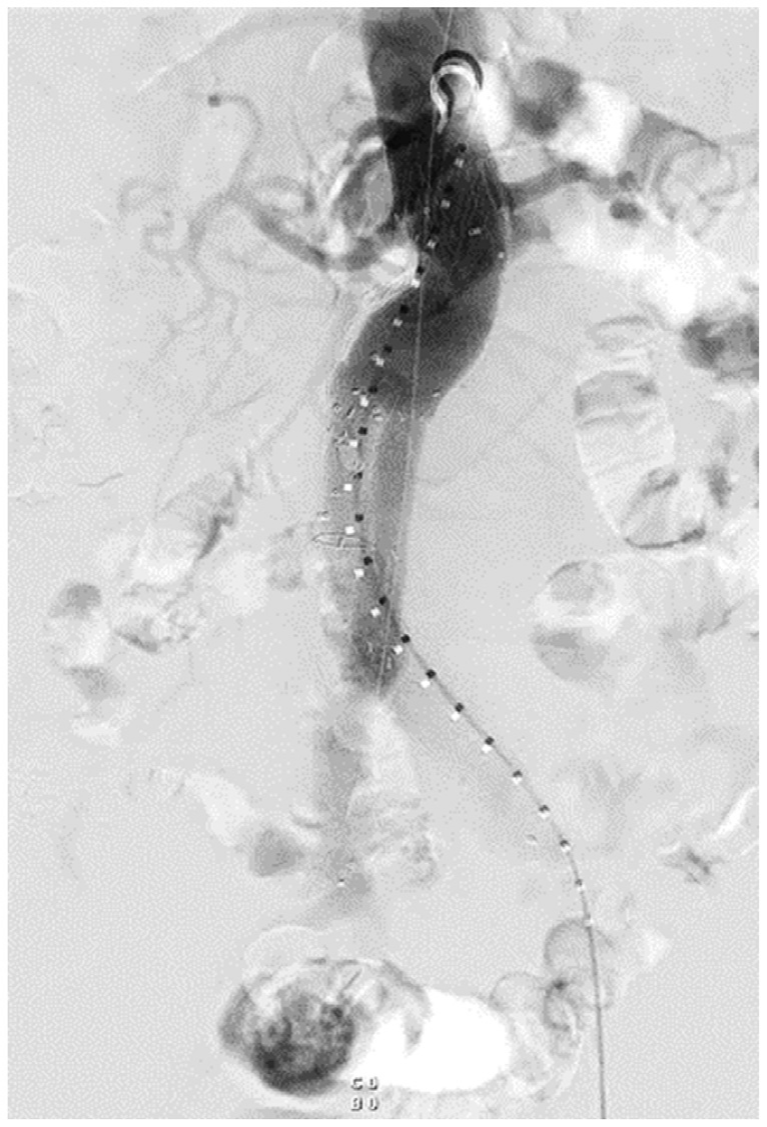
Completion angiography demonstrated widely patent bilateral iliac limbs with complete exclusion of the aneurysm sac.

**Fig 6:**
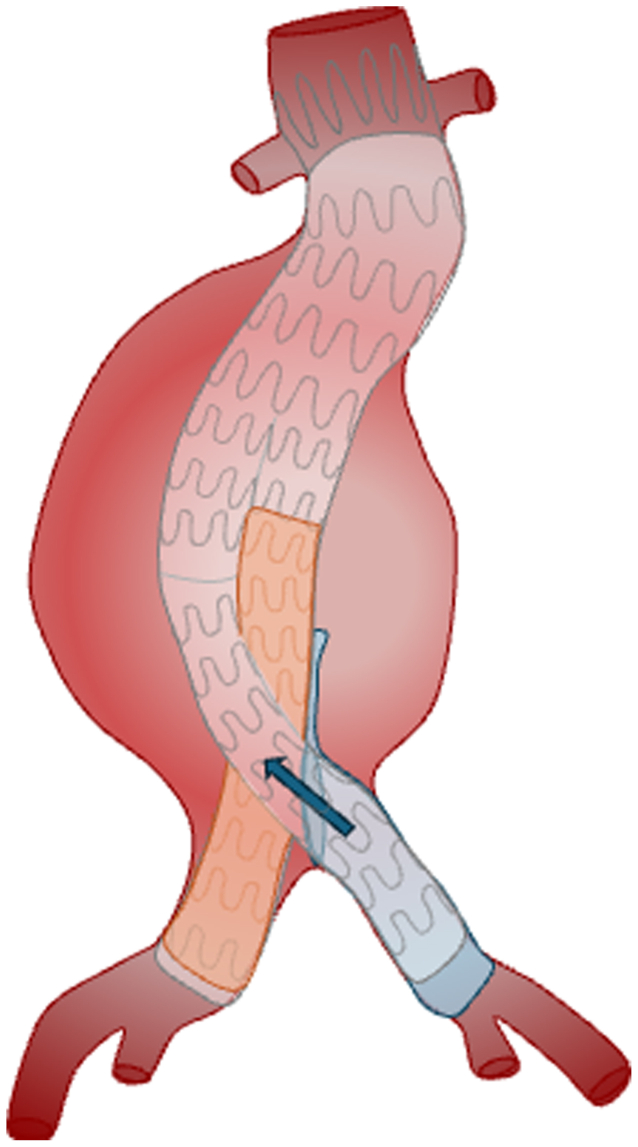
A schematic diagram depicting the different components used to salvage the misdeployed right iliac limb. The original right iliac limb is depicted in *light blue* and can be seen deployed outside of the contralateral gate. A fenestration (*blue circle*) was created through the misdeployed right iliac limb into the aneurysm sac. This allowed for cannulation of the empty gate via the fenestration (*blue arrow*), and a new iliac limb was deployed through the fenestration into the pre-existing right iliac extension. A balloon expandable stent (*orange*) was deployed in the proximal left iliac limb to crush the misplaced right iliac limb within the gate.

ISLF was also used in a 73-year-old male with a 5.2-cm infrarenal AAA who underwent EVAR at an outside hospital. Postoperative CT angiography (CTA) demonstrated an endoleak originating near the flow divider. He was referred to our clinic for possible endoleak embolization. Close inspection of the CTA revealed that both iliac limbs had been deployed in the contralateral gate, causing a type III endoleak from the unoccupied ipsilateral leg off the EVAR main body. The suspicion of limb misdeployment was confirmed intraoperatively on live fluoroscopy images (Fig 4), digital subtraction angiography, and IVUS. To address this error without open conversion, a fenestration was created through the misdeployed right iliac limb into the aortic aneurysm sac using the 0.9-mm Excimer laser utilizing a similar technique to the aforementioned case. With wire access into the aneurysm sac, the opening to the ipsilateral leg off the bifurcated main body was selectively catheterized, and a new iliac limb was deployed through the newly created fenestration into the pre-existing right iliac extension. A large balloon expandable stent (VBX, W.L Gore & Associates) was deployed in the proximal left iliac limb to crush the misplaced right limb within the gate. Completion angiogram (Fig 5) and postoperative CTA demonstrated widely patent bilateral iliac limbs with complete exclusion of the aneurysm sac. A schematic diagram depicting the relative positions of the different components used in the salvage of this misplaced right limb is shown in Fig 6.

## Discussion

This case series presents an innovative approach using ISLF to salvage misdeployed limbs during EVAR, thereby avoiding the need for open conversion or additional bypass procedures. The ability to salvage misdeployed limbs using minimally invasive techniques such as ISLF not only mitigates procedural risks but also reduces postoperative morbidity and the prolonged hospitalization associated with open procedures. Specifically, open conversion is associated with high rates of early readmission and reintervention, and high rates of cardiac, renal, and pulmonary complications.^8^^,^^9^ Thus, the development of endovascular techniques to manage EVAR complications is essential.

Although the literature describes multiple techniques that could be utilized to avoid failed contralateral gate catheterization, such as the pigtail catheter rotation test, visualization of the inside of the gate on IVUS, and lateral fluoroscopy images to confirm gate cannulation, research regarding endovascular salvage techniques for this procedural error remains sparse. Case reports have described the use of parallel grafting,^10^ repositioning a misdeployed stent,^11^ pushing the misdeployed stent into the aneurysm sac,^12^ and crushing of a misdeployed stent^13^ to address this mistake endovascularly. However, there is no consensus on the most efficacious or durable endovascular approach for EVAR salvage of misdeployed iliac limbs, given its rarity.^12^ Gate cannulation confirmation must rely on different techniques, including balloon occlusion and deformation of the gate, rotation of a pigtail or similar catheter inside the endograft, and IVUS. Angiography must be used to confirm access to the main body of the bifurcated endograft. A combination of these techniques should be used, particularly when in doubt about successful gate cannulation. Our experience suggests that ISLF represents a promising adjunct in the armamentarium of endovascular techniques available during complex EVAR with excellent short-term results with no additional morbidity for the patient. More importantly, ISLF of the misplaced leg endograft and catheterization of the contralateral gate with relining of the iliac limbs restore aortoiliac anatomic continuity and eventually render an intact EVAR.

Despite the successful outcomes observed in our cases, the safety and effectiveness of ISLF for salvaging limb misdeployment requires additional investigation to ensure its safety and durability. Moreover, the technical nuances for ISLF during EVAR remain to be defined. Of importance, adjunct imaging techniques, such as the use of three-dimensional CTA fusion, cone-beam CT to assess the misplaced limb in relation to the aneurysm sac, and IVUS are critical to steer the ISLF catheter in the right direction and avoid aortic injuries and rupture. Factors including anatomical suitability, endograft type, and operator expertise are likely to influence procedural success. However, with the widespread adoption of ISLF for subclavian, hypogastric, and renovascular revascularization, surgeon familiarity and availability of the necessary instruments make this technique particularly attractive and easily adoptable.

## Conclusions

EVAR limb misdeployment can be a devastating complication that typically requires open conversion. ISLF through the misdeployed limb is a novel bail-out endovascular technique that circumvents the need for open conversion or a femoro-femoral bypass.

## Funding

None.

## Disclosures

M.S.B. and C.H.T. have been consultants for and received research support from Cook Medical Inc, W. L. Gore & Associates, Inc, and Phillips Healthcare. M.L.K. has been a consultant for W. L. Gore & Associates, Inc.
